# Pericarditis

**Published:** 1842-07-02

**Authors:** 


### CLINICAL REPORTS.

*Blockley Hospital—Service of W. W. Gerhard, M. D.*

Reported by M. W. Wilson, M. D., Resident Physician.

*Pericarditis.*—Two cases come under our notice,—one white man and one black. In the first the disease supervened on an attack of acute articular rheumatism. The case was a mild one, and the patient convalesced in about ten days. The case presented nothing worthy of remark ; it was but an instance of the connection of pericarditis with rheumatism, a rule now so well established, that if a patient has the latter disease in a severe form, we know that pericarditis will accompany it in the large majority of cases. As in most cases of rheumatic pericarditis, no treatment was especially directed to the heart ; the general treatment is quite sufficient.

Theodore Joseph, ætat. 28, coloured man, entered the wards on the 19th of April.

[At the time of entrance of the patient he was too feeble, and his intellect too dull, to obtain any accurate history of his case.]

22d. *Present state.* Head considerably elevated ; dyspnœa ; respiration 58, very irregular, short, chiefly abdominal ; pulse 84, slightly thrilling and irregular, not intermittent. Cough short and dry ; expectoration viscid. Heat of skin moderate; tongue large and white; bowels open last night. Impulse of heart feeble, slightly thrilling and diffused. Percussion dull over second rib for an inch down the sternum, then perfect flatness, which extends to below the nipple, and a little anterior to the posterior portion of axilla. Right side, flatness of lower half. Respiration vesicular under left clavicle; sounds of heart feeble; both sounds a little harsh; various modification of the friction sound. On the right side, where dulness exists, crepitant rhonchus. Posteriorly on the right side, upper lobes, crepitant rhonchus and imperfect bronchial respiration; lower lobe, bronchial respiration. Left side. crepitant rhonchus abundant also; extent nearly as on right side. Ordered four dry and four scarified cups between the shoulders.

℞ Mass. Hydrarg. gr. j.; Ipecac. Pul., gr. ss., M. q. h. s. ℞ Ammon. Carb., gr. iiss., q. h. 2. ℞ Vini ℥iv. per diem.

Evening. Pulse 92, more full and strong; respiration. Ordered six scarified cups to chest, posteriorly.

23d. Improved appearance; pulse 92, more full and soft; respiration 46, still irregular; sounds of heart clearer, friction sound doubtful; respiration above the heart clear. Right side, slight bronchial respiration at root, and slight crepitant rhonchus in lower lobe. Left side, crepitant rhonchus, abundant and loose in lower lobe, loud bronchial respiration only at root. Percussion clear on right side; on left side as yesterday. Ordered six scarified cups to chest. Continue treatment. Evening, pulse 104; respiration 56.

24th. Slept well; bowels open twice during the night. Pulse 86, soft and nearly regular; respiration 48. Continue treatment.

25th. Decubitus horizontal; pulse 92, soft and regular; respiration as yesterday.

Impulse of heart stronger, and sounds clearer; both sounds heard; a faint bellows murmur with first, no creaking.

Respiration vesicular and puerile in upper lobe; bronchial at root, and crepitant rhonchus at the bottom of lower lobe. Right side, posteriorly, feeble and harsh. Rude at the summit, anteriorly. Percussion less dull on left side. Ordered four cut cups posteriorly to chest. Continue treatment. Evening, pulse 100; respiration 48.

26th. Slept none last night. Pulse 96; respiration 48, more regular. Appetite not so good.

27th. Pulse 104, rather feeble and thrilling; respiration 48, high, irregular; expectoration slightly rusty, rare, very viscid; complains of more dyspnœa; respiration on left side still bronchial. Right side vesicular throughout; sounds of heart feeble, impulse strong, action jerking; very slight creaking sound over heart.

Flatness on percussion over præcordial region about the same as before; left side posteriorly flat throughout the greater portion. Ordered two scarified cups and three dry ones to chest posteriorly. Mercurial increased one half.

28th. Pulse 104; characters as yesterday. Respiration 44, high, irregular; expectoration more copious.

29th. Respiration still frequent, 56 after excitement; pulse 120, small and feeble; intellect clear; cough clear and loose; expectoration slightly muco-purulent.

℞. Ammon. Carb. ʒj.

Syr. Senegæ, ℥j.

Mucil. G. Acac. q. s. ℥vj.

M. ℥ss. q. h. s.

Evening. Pulse very small and feeble, 104.

Respiration very laboured, 54; extremities cold. Ordered sinapisms to extremities, and

℞. Ol. Terebinth. ℥ss.

Lac. Assafœt. ℥j. per anum.

Died during the night.

*Autopsy twenty-four hours after Death.*

Pericardium contained two quarts of purulent fluid; on its interior surface it exhibited patches of bright red injection, corresponding with the right ventricle and base of the heart; the whole covered with tolerably firm lymph. Heart also covered with lymph, and presents spots of similar redness, corresponding with the redness of pericardium. Parietes of left ventricle somewhat thickened, (not measured.) Right auricle very much dilated. Valves healthy, without redness or lymph. Extensive adhesions of both pleura.

*Lungs.*—Upper lobe of left granulated; lower lobe has some purulent infiltration.

*Right lung* congested; middle lobe granulated; lower lobe infiltrated with purulent matter like the left.

*Liver* considerably enlarged and pale, scarcely fatty.

*Kidneys.*—Commencing granulations in the cortical substance.

[*Remarks.*—The patient had probably been ill two or three weeks before his admission, without assistance of any kind. The dyspnœa was excessive—fifty-eight inspirations in the minute—while the pulse was only eighty-four. This excessive frequency of respiration is in itself almost always a mortal sign, but when it is conjoined with a pulse which is very nearly of the natural frequency, the prognosis cannot well be more unfavourable. The physical signs of pericarditis with effusion, were well developed in this case: the action of the heart was enfeebled by the large mass of liquid, which did not, however, at first prevent the friction sound from being developed. The alterations of the sounds of the heart were extremely slight, which was readily explained from the absence of endocarditis: confirming the rule, that simple pericarditis, with effusion, produces in most cases some alteration of the sounds of the heart, but only to a slight degree. When there is much rasping, or a strong bellows sound, it may be regarded as nearly conclusive evidence of endocarditis. Death took place from the gradual enfeebling of the circulation, from the mass of liquid in the pericardium.

The treatment would probably have been successful in this case, if the patient had not entered at so advanced a period. He evidently improved under the cups, which were the only depletory measures we could employ, but had not sufficient powers of reaction to repair the lesions which were already formed. It was a matter of doubt whether blistering would not have answered a better purpose; cups were, however, preferred, on account of the extreme dyspnœa, and the preservation of a tolerably forcible pulse: they were repeated on account of the relief which for a time followed their use. The mercurials were not used long enough to produce any sensible impression.—W. W. G.]

